# West African Ebola epidemic: lessons and a call to action

**DOI:** 10.11694/pamj.supp.2015.22.1.6040

**Published:** 2015-10-10

**Authors:** Donne Kofi Ameme

**Affiliations:** 1 Ghana Field Epidemiology and Laboratory Training Programme (GFELTP), School of Public Health, University of Ghana, P.O. Box LG 13, Accra, Ghana

**Keywords:** Ebola, West Africa, lessons

## Abstract

The current Ebola epidemic in West Africa is unprecedented in terms of magnitude and spread. A year after the index case had been identified in a remote village in Guinea, over 17,000 cases and 6,000 deaths were reported in Africa and beyond. Many interventions have been implemented but the outbreak rages on. This paper examines key gaps in the interventions and calls for evidence-based actions to reverse the trend and prevent future epidemics of this proportion.

## To the editors of the Pan African Medical Journal

The ongoing Ebola virus disease (EVD) epidemic in West Africa is on record as the worst in history both in magnitude and geographic spread [1, 2] with the number of cases and deaths exceeding that recorded in all previous outbreaks combined. As at December 7, 2014, a year after the index case had been reported, 17,942 cases with 6,388 deaths were recorded [3]. The disease and its implications transcend the borders of West Africa. Though the trajectory of the epidemic has been predicted by various models [4, 5], a firm deadline of when the outbreak will be over is still elusive underscoring the need for rigorous implementation of control measures. Whilst intensifying response, critical introspection becomes imperative as a basis for remedial actions. Overarching among gaps in the response is the much recognized Africa's dysfunctional health systems [2, 6]. The EVD outbreak has been blamed on poor infrastructure, lack of competent health workforce and weak surveillance systems that are not linked to effective response. Next to this is the delay in receiving international support. Once the international community was notified of the epidemic, international collaboration was the next logical expectation. However, there was delay in international response which has been lamented and tagged as sluggish and fragmented [7, 8]. Swift and decisive action could have averted what now stands as a looming catastrophe. Adopting drastic ineffective measures of military led mass quarantine and cordoning off of communities [9] instead of prioritizing case identification, case management, contact tracing and effective preventive measures which underpinned previous control strategies [2], proved counterproductive. The result has been mistrust between communities and their leaders, fuelled by the fragile political landscape created by years of political instability in the most affected countries. Social engagement and community mobilization efforts were undermined as a result.

Another lesson is the lack of preparation exemplified by the most affected countries. This brings to the fore the Nigerian bench mark experience [10]: the model for all at-risk countries. The introduction of the disease to Lagos, a densely populated city, sparked pessimistic projections of imminent disaster. However, implementation of rapid response strategies by leveraging on available public health resources such as a well-trained workforce and a well-coordinated Emergency Operation Centre (EOC) birthed from an Incident Management System (IMS) analogous to that used in the polio eradication program proved rewarding [10]. This approach, albeit with some challenges was missing in the intense-transmission countries: Guinea, Liberia and Sierra Leone. In addition to these are deep-seated cultural practices and resistance to change that are enabling the skyrocketing statistics of EVD cases being churned out from these countries [3]. The disease that thrives on human affection and the African's love for the dead appears to be relishing the cultural practices of hugging, handshaking and unsafe burial practices. Affected communities are averse to disposal of the remains of their loved ones by health workers without them having the opportunity to pay their last respect. Families continue to hide and nurse their sick relations at home contrary to recommendations. These create fertile grounds for the disease to flourish. Undoubtedly, this outbreak when eventually brought under control will leave Africa with valuable lessons because of the paradox of the many preventable deaths caused by a preventable disease. In addition will be relatively stronger health systems or at least, a dire need for them. Going forward, nations have a decision to make: political actors must create the congenial environment necessary to reverse the tide and prevent similar events from getting out of proportion in future. Countries with or without the disease will have to develop functional preparedness and response plans, which run on an EOC/IMS structure. Leadership must emerge from the national EOC/IMS with regular communication based on trust and transparency. National and sub-national capacity must be harnessed through investing in training and resourcing of local public health workforce [6] that can be mobilized within the shortest possible time. Trained workforce must be strategically positioned with clearly defined roles for seamless integration into established incident management frameworks should the need arise. Regular simulation exercises must be conducted to reinforce readiness. National surveillance activities should not take a business-as-usual approach but must evolve into robust systems augmented with cutting edge information technology in line with best practices. Innovation and the search for novel diagnostics and vaccines, though welcome priorities, should in no way detract from doing what works at the moment. Efforts must be focused on complementing these advances with evidence-informed strategies that work rather than adopting unproven standalone measures that cause backlashes [9]. We must document extensively the successes and how not to do things.

## Conclusion

The call to political commitment and leadership in investing in strong sustainable health systems staffed with well-trained, well-paid and ready to be deployed workforce as well as strong partnerships is perhaps the most important message that offers bright prospects of averting future epidemics of similar magnitude.
